# High Prevalence of Posterior Polymorphous Corneal Dystrophy in the Czech Republic; Linkage Disequilibrium Mapping and Dating an Ancestral Mutation

**DOI:** 10.1371/journal.pone.0045495

**Published:** 2012-09-25

**Authors:** Petra Liskova, Rhian Gwilliam, Martin Filipec, Katerina Jirsova, Stanislava Reinstein Merjava, Panos Deloukas, Tom R. Webb, Shomi S. Bhattacharya, Neil D. Ebenezer, Alex G. Morris, Alison J. Hardcastle

**Affiliations:** 1 Laboratory of the Biology and Pathology of the Eye, Institute of Inherited Metabolic Diseases, First Faculty of Medicine, Charles University in Prague and General University Hospital in Prague, Prague, Czech Republic; 2 Department of Ophthalmology, First Faculty of Medicine, Charles University in Prague and General University Hospital in Prague, Prague, Czech Republic; 3 UCL Institute of Ophthalmology, London, United Kingdom; 4 Wellcome Trust Sanger Institute, Hinxton, United Kingdom; 5 European Eye Clinic Lexum, Prague, Czech Republic; 6 Division of Brain Sciences, Hammersmith Hospital, Imperial College, London, United Kingdom; Innsbruck Medical University, Austria

## Abstract

Posterior polymorphous corneal dystrophy (PPCD) is a rare autosomal dominant genetically heterogeneous disorder. Nineteen Czech PPCD pedigrees with 113 affected family members were identified, and 17 of these kindreds were genotyped for markers on chromosome 20p12.1- 20q12. Comparison of haplotypes in 81 affected members, 20 unaffected first degree relatives and 13 spouses, as well as 55 unrelated controls, supported the hypothesis of a shared ancestor in 12 families originating from one geographic location. In 38 affected individuals from nine of these pedigrees, a common haplotype was observed between D20S48 and D20S107 spanning approximately 23 Mb, demonstrating segregation of disease with the PPCD1 locus. This haplotype was not detected in 110 ethnically matched control chromosomes. Within the common founder haplotype, a core mini-haplotype was detected for D20S605, D20S182 and M189K2 in all 67 affected members from families 1–12, however alleles representing the core mini-haplotype were also detected in population matched controls. The most likely location of the responsible gene within the disease interval, and estimated mutational age, were inferred by linkage disequilibrium mapping (DMLE+2.3). The appearance of a disease-causing mutation was dated between 64–133 generations. The inferred ancestral locus carrying a PPCD1 disease-causing variant within the disease interval spans 60 Kb on 20p11.23, which contains a single known protein coding gene, *ZNF133*. However, direct sequence analysis of coding and untranslated exons did not reveal a potential pathogenic mutation. Microdeletion or duplication was also excluded by comparative genomic hybridization using a dense chromosome 20 specific array. Geographical origin, haplotype and statistical analysis suggest that in 14 unrelated families an as yet undiscovered mutation on 20p11.23 was inherited from a common ancestor. Prevalence of PPCD in the Czech Republic appears to be the highest worldwide and our data suggests that at least one other novel locus for PPCD also exists.

## Introduction

Posterior polymorphous corneal dystrophy (PPCD) is a rare bilateral disorder transmitted as an autosomal dominant trait. Clinically PPCD is characterized by vesicles, bands and polymorphous opacities with pathology at the level of Descemet membrane and the corneal endothelium. Peripheral anterior iris adhesions, iris atrophy, pupillary ectropion and corectopia may also develop. Occasional severe visual disability results from secondary glaucoma or corneal edema [1]–[3]. On ultrastructural examination, corneal endothelial cells show fibroblastic and epithelial-like transformation [4]–[7].

The genetic heterogeneity of PPCD is currently known to be represented by three loci on chromosomes 20, 1, and 10 [8]–[11]. PPCD1 (MIM ID #122000) is located on 20p11.21. Mutation of the visual system homeobox gene 1 (*VSX1*; MIM ID #605020) within this locus was reported as disease-causing in a few PPCD cases [12], [13]. PPCD2 (MIM ID #609140) is caused by mutation of the alpha-2 chain of type VIII collagen gene (*COL8A2*; MIM ID #120252) located on 1p34.3-p32.3, however only one disease-causing mutation in one family has been described to date [9]. Mutations in the zinc finger E-box binding homeobox 1 gene (*ZEB1*; MIM ID #189909) mapping to chromosome 10p11.2 were identified as disease-causing in PPCD3 (MIM ID #609141) and it has been estimated that pathogenic changes within this gene account for approximately 25% of all PPCD cases, with a range from 9%–45% depending on population studied [14]–[16]. We have previously shown that in two Czech families PPCD is linked to the short arm of chromosome 20, flanked by the markers D20S48 and D20S139 [11]. Exclusion of *VSX1* from this genetic interval by a linkage study, and lack of disease-causing changes, implies that an as yet undiscovered gene is causative for PPCD1 [11].

Families affected by rare dominantly inherited disorders are often unrelated, however occasionally they share a chromosomal genomic region implying that the pathogenic mutation arose in a common ancestor [17].

In this study we observed that PPCD in the Czech Republic appears to have a remarkably high prevalence. A total of 19 Czech PPCD families, including two previously linked pedigrees [11], were ascertained and members of 17 pedigrees were genotyped for microsatellite markers spanning a region from 20p12.1 to 20q12. We correlated the observed haplotypes with geographical origin of the eldest family member known to suffer from the disorder and demonstrate that the high prevalence of PPCD in the Czech Republic is due to a common founder.

## Materials and Methods

### Patients

The study was approved by the Ethics Committee of General University Hospital in Prague, Czech Republic and conformed to the tenets of the Declaration of Helsinki. All participants signed an informed consent prior to inclusion into the study.

Subjects from 19 Czech pedigrees with familial PPCD were examined between the years 1995–2010 in the Department of Ophthalmology of the First Faculty of Medicine, Charles University in Prague. Ophthalmologic assessment included visual acuity, slit lamp examination, intraocular pressure measurements and specular microscopy using Noncon ROBO Pachy SP-9000 (Konan Medical Inc, Tokyo). Diagnosis of PPCD was based on positive family history and the presence of vesicles and polymorphic opacities at the level of Descemet membrane and the corneal endothelium. Pedigrees were drawn and residency within the Czech Republic of the eldest family member known to suffer from PPCD was noted. Geographic origin of the families was plotted on a map.

### Genotyping and Haplotype Analysis

DNA was isolated from venous blood samples using the Nucleon III BACC3 genomic DNA extraction kit according to manufacturer’s instructions (GE Healthcare, UK). Genotyping was performed using 11 polymorphic microsatellite markers on chromosome 20 which were fluorescently labeled and amplified by polymerase chain reaction (PCR). Ten microsatellites were commercially available: D20S98, D20S118, D20S114, D20S48, D20S605, D20S182, D20S139, D20S190, D20S106 and D20S107 (Invitrogen, Paisley, UK). A dinucleotide marker used in this study, M189K21, was reported previously [11]. Amplification was performed in 25 µl reaction volumes. Markers were run on an ABI 3100 and analyzed using Genescan and Genotyper software (Applied Biosystems, Foster City, CA).

To investigate the possibility of a common lineage, haplotypes of affected individuals were constructed based on segregation within the families, and then compared between families. In order to calculate allele frequencies and haplotype frequencies in the population, 55 unrelated Czech population matched controls (110 chromosomes) were also genotyped for each marker.

### Analysis of the Disease Gene Location and Age of the Mutation

To infer the location of a gene responsible for PPCD1 in the population studied and to estimate the age of the mutation (i.e. the time elapsed since the appearance of the common ancestor in the population) DMLE+ (Disease Mapping using Linkage disequilibrium) version 2.3 (www.dmle.org) was used. The program DMLE+ uses Bayesian estimates of the location of a gene with a mutation affecting a discrete (disease) trait based on the observed linkage disequilibrium at multiple genetic markers. Other parameters are also estimated, such as mutation age [18], [19].

Input for this analysis was the common PPCD genotypes of affected individuals originating from the same geographic location, and the haplotypes of population matched control individuals. Prior to DMLE+2.3 analysis, haplotypes of unrelated controls were predicted using the PHASE program [20], [21]. Genetic distances of individual markers used in the analysis were taken from the Marshfield genetic map (http://research.marshfieldclinic.org/genetics/GeneticResearch/compMaps.asp). For markers which do not appear on this genetic map (D20S98, D20S48, M189K21, D20S139) the distance in cM was estimated empirically by interpolation using the standard curve of physical distance. Since DMLE+2.3 does not accept identical distances for more than one marker, where the position of two markers obtained from the Marshfield genetic map was identical (i.e. D20S118, D20S114, D20S605, D20S182) position was recalculated using the standard curve as above (see Supporting Information Table S1). The following variable values were used in the DMLE+2.3 analysis: population growth rate of 0.95. This was calculated from an estimated population size of 100,000 inhabitants 2000 years ago and current population size of 6,700,000 inhabitants living in the historic regions of Bohemia that have been historically compact. The estimated frequency of the disease in the population was set at 0.001.

### Screening the *ZNF133* Gene

Two probands from families 1 and 2, previously shown to be linked to 20p11.2 [11], as well as one unaffected first-degree relative from family 2 were screened. Amplification was carried out in a 25 µl reaction containing 12.5 µl of ReddyMixTM PCR master mix (ABgene Limited, Epsom, UK), with 50 pmoles gene-specific primers (see Supporting Information Table S2) and approximately 50 ng of genomic DNA. The PCR amplicons were purified and sequenced on both strands as previously described [22]. Nucleotide sequences were compared with the published zinc finger protein 133 (*ZNF133;* MIM ID #604075) reference sequence (NM_003434 and NM_001083330). Both coding and untranslated regions as well as intron-exon boundaries were screened.

### Comparative Genomic Hybridisation

Array comparative genomic analysis (CGH) was used to evaluate DNA copy number variation (CNV) on chromosome 20 (NimbleGen, Berlin, Germany) with a median probe spacing of 134 bp. Patient DNA from family 1, previously shown to be linked to 20p11.2 [11], was labeled with Cy-3 and a reference sex matched DNA sample was labeled with Cy5. Array construction, labeling, hybridisation, and normalisation were performed by NimbleGen. The data were visualised and analysed using SignalMap software (NimbleGen).

## Results

**Figure 1:**
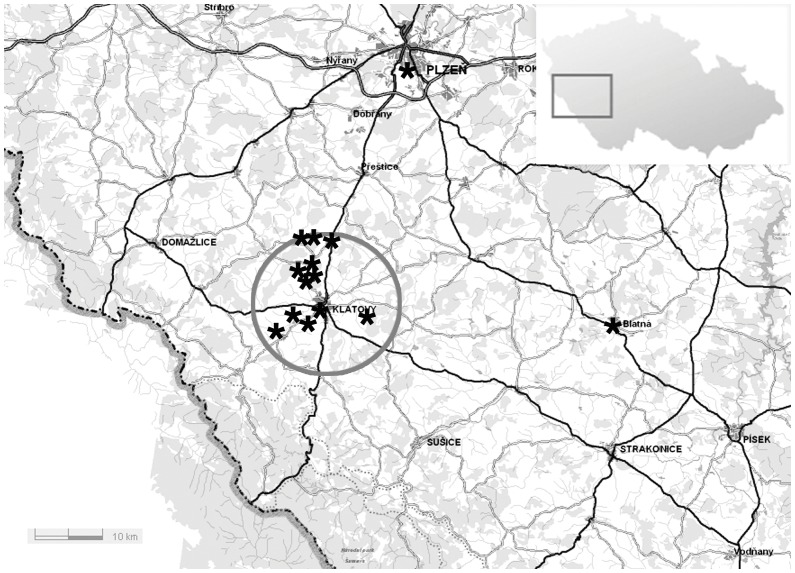
Geographical origin of families with posterior polymorphous corneal dystrophy within the southwestern part of the Czech Republic. The geographical origin of the eldest members of each family is indicated by *. Twelve of the families, of which ten were genotyped and shown to share common haplotype, can be traced to a region of 13 km radius around the town of Klatovy. Two other genotyped families with the common full haplotype spanning over 23 Mb originate from an approximate 40 km radius from Klatovy, however knowledge of the place of origin only extended to three generations in both families.

Clinical ascertainment demonstrated bilateral corneal changes consistent with the diagnosis of PPCD in 113 subjects (48 males, 65 females) from 19 families. None of the PPCD families were known to be related to one another. In all families PPCD was consistent with autosomal dominant inheritance pattern, with incomplete penetrance noted for family 18 and 19 [23]. Of these, 32 patients were from two large pedigrees (families 1 and 2) previously linked to the short arm of chromosome 20 [11]. Families 1–14 originated from the western or southwestern part of the Czech Republic, 12 of which were from a small region of approximately 13 km radius around the town of Klatovy (Figure 1) with an estimated current population of 30,000 inhabitants. Out of the 19 families identified, 81 affected individuals from 17 families were genotyped, including members of the two families in which linkage analysis was previously performed [11]. Table 1 summarizes the number of individuals genotyped for each family, their clinical status, molecular genetic findings and geographical origin within the Czech Republic.

**Table 1:** Summary of posterior polymorphous corneal dystrophy study families and subjects.

Family	Examinedaffected	Genotypedaffected	Genotyped unaffected	Genotypedspouses	Incomplete penetrance	Number of affected individuals with common haplotype on 20p12.1- 20q12/core mini-haplotype	Geographical origin
1	15	14	7	5	N	10/14	Southwest
2	16	14	6	5	N	6/14	Southwest
3	8	7	0	1	N	6/7	Southwest
4	5	5	1	0	N	2/5	Southwest
5	10	7	2	1	N	6/7	West
6	9	6	2	0	N	2/6	Southwest
7	4	4	0	0	N	3/4	Southwest
8	5	4	0	0	N	2/4	Southwest
9	3	2	0	0	N	0/2	Southwest
10	2	2	0	0	N	0/2	Southwest
11	2	1	1	0	N	0/1	Southwest
12	1	1	0	0	N	1/1	Southwest
13	6	0	0	0	N	Not performed	Southwest
14	2	0	0	0	N	Not performed	Southwest
15	16	7	1	0	N	0/0	Central
						(Segregation with 20p12.1- 20q12 excluded byhaplotype analysis)	
						No mutation in *VSX1*, *COL8A2* or *ZEB1*	
16	2	2	0	0	N	0/0	Northeast
						No mutation in *VSX1*, *COL8A2* or *ZEB1*	
17	3	1	0	0	N	0/0	East
						No mutation in *VSX1*, *COL8A2* or *ZEB1*	
18	3	3	0	1	Y	*ZEB1* mutation identified	North
						No mutation in *VSX1* or *COL8A2*	
19	1	1	0	0	Y	*ZEB1* mutation identified	Central
						No mutation in *VSX1* or *COL8A2*	
Total	113	81	20	13			

Number of phenotyped and genotyped affected family members, unaffected first degree relatives and spouses included in this study. Presence of shared haplotype across 20p12.1- 20q12 spanning over 23 Mb as well as the core mini-haplotype at 20p12.1-20p11.23 in affected individuals, previous molecular genetic analysis and geographical origin within the Czech Republic of the eldest family member known to be affected is also shown.

N = No, Y = yes.

Linkage analysis for families 1 and 2 was reported in Gwilliam *et al*. [11]. Results of previous candidate gene screening in families 15–19 has been reported in Liskova *et al*. [23].

Analysis of genetic markers on 20p12.1- 20q12 revealed a common haplotype spanning a region of at least 23 Mb surrounding the centromere and encompassing both the short and long arm of chromosome 20. This full common haplotype was detected in 16 affected members of the two previously linked PPCD1 families (1 and 2) and also in 22 affected members from families 3–8 and 12 (see Supporting Information Table S3). None of the 55 population matched controls had a combination of markers that would allow for reconstruction of this full haplotype. The founder haplotype segregated with disease in families with more than one genotyped affected individual. In all 67 affected members from families 1–12 a core shared mini-haplotype was detected for D20S605, D20S182 and M189K2. The boundary of this haplotype was defined by recombinations with flanking markers D20S48 and D20S139 as less than 2.4 Mb (see Supporting Information Table S3). However, alleles representing this mini-haplotype were also found in 12 controls out of 55.

In families 15–19, analysis did not reveal the common shared haplotype detected in families clustered from the southwest part of the Czech Republic. In family 15, segregation of genotyped markers on 20p12.1-20q12 with PPCD was not observed suggesting they are not linked to this locus. In family 16, only two affected family members were genotyped and in families 17 and 19 only the proband was available for genotyping, thus assessment of haplotype segregation with disease could not be reliably performed (see Supporting Information Table S3).

**Figure 2:**
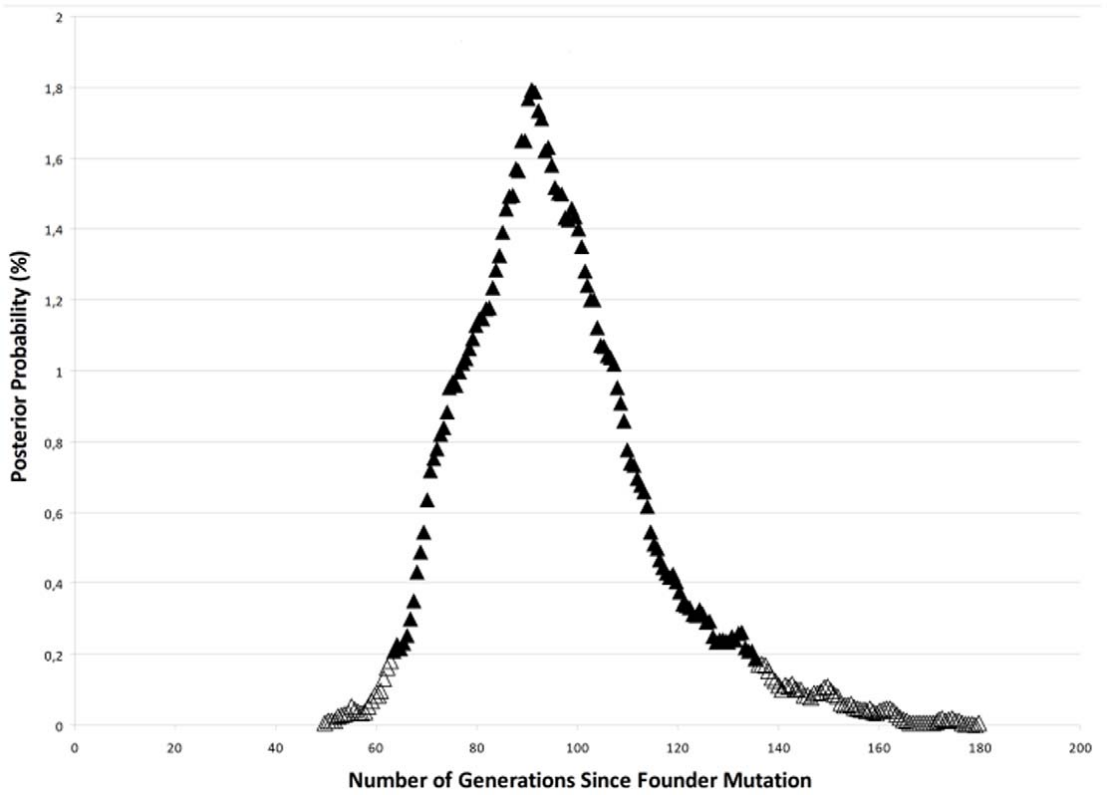
Estimated age for posterior polymorphous corneal dystrophy linked to 20p12.1-20p11.23 in Czech patients. Maximum probability represented by 5% significance interval shown as filled triangles was found at 90 generations (range 64 to 133), i.e. 1800 years assuming a 20-year generation time.

**Figure 3:**
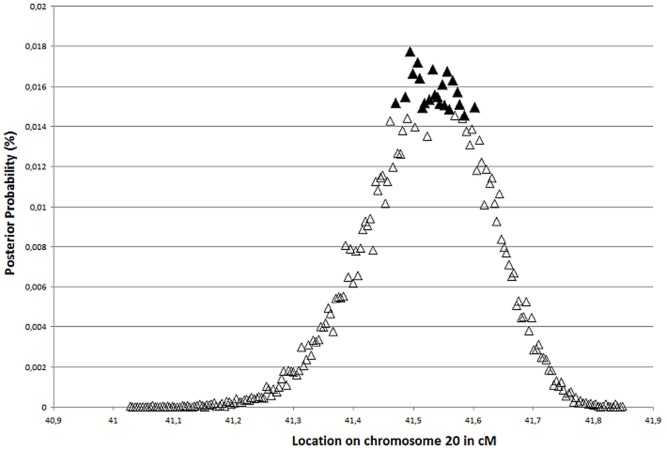
Predicted location of the putative mutation causing posterior polymorphous corneal dystrophy on 20p11.23. Filled triangles show 5% significance level of disease mutation location on chromosome 20 in cM; a 60 kb region between markers D20S182 and M189K21.

Analysis of the haplotypes using DMLE+2.3 yielded an estimate that families 1–12 had a common ancestor originating between 64–133 generations ago, with maximum posterior probability at 90 generations; i.e. 1800 years assuming a 20-year generation time or 2250 years with a 25-year generation time (Figure 2). The mutation locator identified the potential locus harboring the ancestral disease-causing change between D20S182 and M189K21 (Figure 3) with 95% confidence. This interval corresponds to a physical distance of only 60 Kb which contains one known protein coding gene; *ZNF133*.

The *ZNF133* gene was therefore screened by direct sequencing for a mutation in probands from two Czech PPCD1 families (1 and 2), however no potential pathogenic changes in the annotated coding and untranslated regions were identified. Since a deletion or duplication of the *ZNF133* gene would not be detected by PCR amplification and sequencing, we also performed dense chromosome 20 CGH analysis (Nimblegen) to detect any CNVs in an affected individual from family 1. No microdeletions or duplications on 20p12.1-20p11.23 were detected in this patient.

## Discussion

Identification of 113 affected individuals from 19 Czech PPCD families demonstrates, to the best of our knowledge, the highest reported prevalence of PPCD worldwide. Correlated to the population, at least 1 in 100,000 inhabitants in the Czech Republic are affected with PPCD. Because of the relative rarity of this disorder, a founder effect was suspected as the most likely explanation.

In this study we show that 38 affected individuals from nine families, including two previously reported families [11], who originate from a particular region within the Czech Republic display a common disease-associated haplotype across a region of at least 23 Mb on chromosome 20. This particular combination of marker alleles was not detected in any of the 55 ethnically matched controls (110 chromosomes) and is therefore markedly over-represented in Czech PPCD families. In addition, 29 affected individuals from 12 families display only part of the disease-associated haplotype, however corroboration of regional clustering also supports the hypothesis that they are descendants of the same ancestor. In two families (13 and 14) affected individuals were not available for genotyping, however because of their geographical origin from the same region as pedigrees 1–12 and the severity of their phenotype, it is likely that they would also share the same common ancestor. Since the information on family origin was based on the eldest member known to clearly suffer from PPCD, we could only trace ancestors back to the last century. Despite the fact that some families originated in nearby villages we were not able to link the pedigrees so they were considered as unrelated for the statistical analyses.

Affected individuals within Czech PPCD1 pedigrees were observed in all subsequent generations implying that the disorder is highly if not fully penetrant, consistent with other studies [8], [24].

Although the fully shared haplotype among members of nine families comprised about 1/3 of chromosome 20 including the centromere and pericentromeric regions, we have calculated that the original mutational event did not occur recently. Since the families originate from one particular geographical area, we assume that they represent a relatively isolated population, which are known to have more extensive linkage disequilibrium than outbred populations [25]. In addition, some regions of genomes are less prone to recombination and large regions of linkage disequilibrium have been shown to occur especially around the centromere [26], [27].

The mutation age was estimated to be 1800 years assuming a 20-year generation time. It remains to be elucidated if the disease-causing variant arose in the Czech Republic and is specific for that particular geographical region or if it is an ancient mutation introduced from elsewhere. Once the PPCD1 causing gene is discovered it will be possible to further explore these hypotheses.

The minimal shared region in families 1–12 was observed between D20S48 and D20S139, which corresponds to the locus delineation by linkage analysis we described previously in Czech families [11]. Yellore *et al.* narrowed the proximal boundary of the PPCD1 genetic interval to D20S182 defining the critical disease physical interval to a 1.8 Mb region, under the assumption that there is no micro-heterogeneity [24]. Despite the fact that the PPCD1 locus has been refined to a relatively small interval, the disease-causing gene still remains to be identified even after next-generation sequence analysis of the entire selectively enriched chromosomal region between markers D20S48 and D20S190 [28].

The strongest candidate disease gene based on our current analysis is *ZNF133* which is a transcriptional repressor containing KRAB box and zinc finger domains [29] with corneal endothelial expression (http://www.corneanet.net/) [30]. Coupled with the fact that mutations in another zinc finger protein are already known to cause PPCD3 [14] we considered *ZNF133* to be the best positional candidate in the refined PPCD1 region, however we did not detect a pathogenic change within currently annotated coding or untranslated regions of this gene. Similarly, no pathogenic variants were observed for *ZNF133* in two other studies using probands from different PPCD families linked to chromosome 20 [28], [31]. A CNV or balanced translocation was also considered, however routine karyotype examination in probands from families 1 and 2, and CGH analysis of an affected member of family 1 did not identify any gross or subtle CNVs at this locus. The lack of identification of a pathogenic mutation suggests that if *ZNF133* is disease-causing, the change might be located in regulatory sequences, as yet unidentified exons or deep within the introns of this gene.

The data presented here, taken together with previous linkage studies, candidate gene screening, targeted genomic next-generation sequencing [8], [11], [25], [28], [31] and our CGH data excluding a CNV, it is perhaps surprising that the causative mutation/gene has not been identified. Potential for locus micro-heterogeneity may be hindering progress. In addition our current functional knowledge of candidate genes in the interval, including *ZNF133,* is very limited such that splice variants and important elements controlling transcription remain undefined. The causative mutations may lie in these uncharacterised upstream regions, exons, and introns or within as yet undiscovered genes on 20p12.1-20p11.23. Independent analysis of each family, by targeted next generation sequencing of the disease interval defined by recombinations within a single large linked family, would circumvent any assumption of locus homogeneity, and may represent the most comprehensive approach, if complemented with Sanger sequencing of gaps and gene expression studies.

Families 15–19 apparently originate from different parts of the country than families 1–14 and these families provided an excellent internal control for our analyses. None of these kindreds had the full consensus haplotype and, with the exception of the proband from family 19, they did not share the minimal core haplotype segment found in all 64 affected individuals from pedigrees 1–12. Based on these observations, it is likely that PPCD in these families is caused by a different mutation or a different gene. In support of this, disease-causing mutations in *ZEB1* were detected in families 18 and 19 [23]. No mutations in coding regions of any currently known PPCD genes were detected in families 15–17 which suggests that a novel PPCD locus may exist [23] (Table 1).

We conclude that in the Czech population *ZEB1* changes account for approximately 4% cases, whereas a disease-causing gene at the 20p12.1-20p11.23 locus is likely to be responsible for more than 80% of all PPCD cases. A recent study by Vincent *et al*. highlights the fact that in some cohorts *ZEB1* accounts for a smaller proportion of cases than originally thought [14]–[16].

Clinical implications of our study lie in the fact that PPCD1 seems to be more severe showing a higher percentage of secondary glaucoma and necessity for keratoplasty than PPCD3, which has a direct impact on patient counseling [32]. Our data suggests that once the PPCD1 gene is identified, it will account for the majority of PPCD in the Czech Republic thus a diagnostic test could be readily developed which would benefit this patient group.

In summary we have demonstrated that the high frequency of PPCD in the Czech Republic is attributable to a founder mutation located on 20p12.1-20p11.23. The disease gene and causative variant have yet to be identified. We anticipate that approximately 80% of PPCD in the Czech population will be attributed to the mutant allele at the locus on chromosome 20p, however it is also clear that other PPCD genes or alleles are implicated as not all families described in this study share a common founder haplotype. Consistent with this finding *ZEB1* mutations were previously identified in two families (18, 19), and lack of the PPCD1 haplotype in two families (16, 17) suggests they may not be associated with the PPCD1 locus. In family 15, the PPCD1 locus is excluded and no mutation was detected in *VSX1, COL8A2* or *ZEB1* suggesting a novel locus may exist.

## Supporting Information

Table S1 **Microsatellite markers used to construct haplotypes.** Microsatellite markers on chromosome 20 and their extrapolated genetic positions used for genotyping in the current study. Physical distances were from Ensembl release 57 and sex-averaged genetic distances were from The Marshfield Comprehensive Human Genetic Map.
(DOC)

Table S2 **Analysis of the** ***ZNF133*** **gene.** Primers used for amplification and sequencing *ZNF133*, their melting temperature (Tm), length of each amplified fragment and variations identified in the proband from family 1 (A), proband from family 2 (B) and first degree relative from family 2 (C) are shown. Exon number corresponds to reference sequence NM_003434.
(DOC)

Table S3 **Identification of a founder haplotype in Czech PPCD families.** Each affected individual is represented by a column, presence of the same allele as the consensus haplotype is indicated by x, and presence of the full common haplotype spanning a region of at least 23 Mb is indicated by o. All 67 affected members from Families 1–12 originating from the same geographic area within the Czech Republic shared a conserved chromosomal region between D20S48 and D20S139 (highlighted in bold). In families 15–19 originating from other parts of the Czech Republic the core haplotype segment between D20S48 and D20S139 was not shared among affected individuals. Affected members from families 13–14 were not available for genotyping.
(DOC)
