# Supplementary material for: High Prevalence of Posterior Polymorphous Corneal Dystrophy in the Czech Republic; Linkage Disequilibrium Mapping and Dating an Ancestral Mutation
Source: PLoS One. 2012 Sep 25;7(9):e45495. doi: 10.1371/journal.pone.0045495 (PMC3458081; doi:10.1371/journal.pone.0045495)
Supplement: Table S1 — Microsatellite markers used to construct haplotypes. Microsatellite markers on chromosome 20 and their extrapolated genetic positions used for genotyping in the current study. Physical distances were from Ensembl release 57 and sex-averaged genetic distances were from The Marshfield Comprehensive Human Genetic Map. (DOC) [file pone.0045495.s001.doc]

**Table S1 Microsatellite markers used to construct haplotypes.** Microsatellite markers on chromosome 20 and their extrapolated genetic positions used for genotyping in the current study. Physical distances were derived from GRCh37/hg19 and sex-averaged genetic distances were from The Marshfield Comprehensive Human Genetic Map.

| **Marker** | **Locus** | **Physical location (Mb)** | **Genetic position (cM)** | **Extrapolated genetic position (cM)** |
| --- | --- | --- | --- | --- |
| D20S98 | 20p12.1 | 15.65 |  | 37.65 |
| D20S118 | 20p12.1 | 17.03 | 39.25 | 39.00 |
| D20S114 | 20p12.1 | 17.26 | 39.25 | 39.25 |
| D20S48 | 20p12.1 | 17.31 |  | 39.35 |
| D20S605 | 20p12.1 | 17.73 | 39.90 | 39.90 |
| D20S182 | 20p12.1 | 17.84 | 39.90 | 40.00 |
| M189K21 | 20p11.23 | 18.39 |  | 41.85 |
| D20S139 | 20p11.23 | 19.65 |  | 45.50 |
| D20S190 | 20p11.22 | 21.31 | 47.52 | 47.52 |
| D20S106 | 20q11.22 | 33.51 | 50.81 | 50.81 |
| D20S107 | 20q12 | 38.88 | 55.74 | 55.74 |
