# Supplementary material for: High Prevalence of Posterior Polymorphous Corneal Dystrophy in the Czech Republic; Linkage Disequilibrium Mapping and Dating an Ancestral Mutation
Source: PLoS One. 2012 Sep 25;7(9):e45495. doi: 10.1371/journal.pone.0045495 (PMC3458081; doi:10.1371/journal.pone.0045495)
Supplement: Table S2 — Analysis of the ZNF133 gene. Primers used for amplification and sequencing ZNF133, their melting temperature (Tm), length of each amplified fragment and variations identified in the proband from family 1 (A), proband from family 2 (B) and first degree relative from family 2 (C) are shown. Exon number corresponds to reference sequence NM_003434. (DOC) [file pone.0045495.s002.doc]

**Table S2 Analysis of the *ZNF133* gene.** Primers used for amplification and sequencing *ZNF133*, their melting temperature (Tm), length of each amplified fragment and variations identified in the proband from family 1 (A), proband from family 2 (B) and first degree relative from family 2 (C) are shown. Exon number corresponds to reference sequence NM_003434.

| **Exon** | **Primers (5’-3’)** | **Tm (°C)** | **Fragment length (bp)** | **Identified variations** |
| --- | --- | --- | --- | --- |
| 1 | GGGAGCTCCTACGTGTTTTATG | 60 | 459 | - |
|  | ATAAGACATCTGGGAGAGGACG | 59 |  |  |
| 2 | GCATCCTTTGGGGTTTCTTT | 60 | 466 | - |
|  | TGCAGAAAGTGATGCTGAGG | 60 |  |  |
| 3 | TGCAAAACCCATGACACAGT | 60 | 408 | rs2252750 G>A |
|  | AGGGTCCTTATTGTCCACCA | 59 |  | heterozygous A,B,C |
| 4 | CTGGATTCTACCAGTAGGTGGC | 60 | 472 | - |
|  | AGCAGAGAGTAGTGAAGCCCTG | 60 |  |  |
| 5 | TTGTGCACATATACCCTGTGTG | 59 | 450 | - |
|  | CCCAAAGGTCAAGTAACAAAGC | 60 |  |  |
| 6 | AGTTCACTGCCCACTCATCC | 60 | 387 | - |
|  | ATCTAAACAGCAGCCCAGGA | 60 |  |  |
| 7A | AGGCTGCCCACTGAGGAC | 61 | 733 | rs2228274 G>A |
|  | TGCACATGTAAGGTTTCTCACC | 60 |  | homozygous A, heterozygous B |
| 7B | TTAACCGGAAGTCAACGCTAAT | 60 | 390 | rs1050475 C>T |
|  | GCTTCTCCTTTGAGTGTGTCCT | 60 |  | homozygous A, heterozygous B |
| 7C | CATCGTGTGCAGTGACTGTG | 60 | 403 |  |
|  | CGTCCTCTGGTGTCTGATGA. | 60 |  |  |
| 7D | AGGACACACTCAAAGGAGAAG | 60 | 575 | rs1050476 T>C |
|  | AGGTGTGACTTTTGGAGAAAGC | 60 |  | homozygous A, heterozygous B |
| 7E | GCTCTAATTACACACAAGCGGG | 61 | 479 |  |
|  | GTGAATGAGGGACACAGGCAC | 63 |  |  |
