# Supplementary material for: High Prevalence of Posterior Polymorphous Corneal Dystrophy in the Czech Republic; Linkage Disequilibrium Mapping and Dating an Ancestral Mutation
Source: PLoS One. 2012 Sep 25;7(9):e45495. doi: 10.1371/journal.pone.0045495 (PMC3458081; doi:10.1371/journal.pone.0045495)
Supplement: Table S3 — Identification of a founder haplotype in Czech PPCD families. Each affected individual is represented by a column, presence of the same allele as the consensus haplotype is indicated by x, and presence of the full common haplotype spanning a region of at least 23 Mb is indicated by o. All 67 affected members from Families 1–12 originating from the same geographic area within the Czech Republic shared a conserved chromosomal region between D20S48 and D20S139 (highlighted in bold). In families 15–19 originating from other parts of the Czech Republic the core haplotype segment between D20S48 and D20S139 was not shared among affected individuals. Affected members from families 13–14 were not available for genotyping. (DOC) [file pone.0045495.s003.doc]

**Table S3 Identification of a founder haplotype in Czech PPCD families.** Each affected individual is represented by a column, presence of the same allele as the consensus haplotype is indicated by x, and presence of the full common haplotype spanning a region of at least 23 Mb is indicated by o. All 67 affected members from Families 1-12 originating from the same geographic area within the Czech Republic shared a conserved chromosomal region between D20S48 and D20S139 (highlighted in bold). Affected members from families 13-14 also having a known affected ancestry in the same geographic region were not available for genotyping. In families 15-17 originating from other parts of the Czech Republic the core haplotype segment between D20S48 and D20S139 was not shared among affected individuals. In families 18 and 19 a *ZEB1* disease-causing variant had been identified and their haplotype is therefore also not shown.

| **Markers** | **Common haplotype in full** | **1** | | | | | | | | | | | | | | **2** | | | | | | | | | | | | | |
| --- | --- | --- | --- | --- | --- | --- | --- | --- | --- | --- | --- | --- | --- | --- | --- | --- | --- | --- | --- | --- | --- | --- | --- | --- | --- | --- | --- | --- | --- |
|  |  | **o** | **o** | **o** | **o** | **o** | **o** | **o** |  |  | **o** |  |  | **o** | **o** | **o** | **o** | **o** | **o** |  |  | **o** |  |  |  |  |  |  | **o** |
| **D20S98** | **5** | x | x | x | x | x | x | x | x | x | x | x | x | x | x | x | x | x | x |  |  | x | x | x |  |  |  |  | x |
| **D20S118** | **3** | x | x | x | x | x | x | x | x | x | x | x | x | x | x | x | x | x | x |  |  | x | x | x | x | x |  | x | x |
| **D20S114** | **1** | x | x | x | x | x | x | x | x | x | x | x | x | x | x | x | x | x | x |  |  | x | x | x | x | x |  | x | x |
| **D20S48** | **3** | x | x | x | x | x | x | x | x | x | x | x | x | x | x | x | x | x | x | x | x | x | x | x | x | x |  | x | x |
| **D20S605** | **2** | **x** | **x** | **x** | **x** | **x** | **x** | **x** | **x** | **x** | **x** | **x** | **x** | **x** | **x** | **x** | **x** | **x** | **x** | **x** | **x** | **x** | **x** | **x** | **x** | **x** | **x** | **x** | **x** |
| **D20S182** | **3** | **x** | **x** | **x** | **x** | **x** | **x** | **x** | **x** | **x** | **x** | **x** | **x** | **x** | **x** | **x** | **x** | **x** | **x** | **x** | **x** | **x** | **x** | **x** | **x** | **x** | **x** | **x** | **x** |
| **M189K21** | **2** | **x** | **x** | **x** | **x** | **x** | **x** | **x** | **x** | **x** | **x** | **x** | **x** | **x** | **x** | **x** | **x** | **x** | **x** | **x** | **x** | **x** | **x** | **x** | **x** | **x** | **x** | **x** | **x** |
| **D20S139** | **5** | x | x | x | x | x | x | x | x | x | x | x | x | x | x | x | x | x | x | x | x | x |  | x | x | x |  | x | x |
| **D20S190** | **5** | x | x | x | x | x | x | x |  | x | x |  |  | x | x | x | x | x | x | x | x | x |  | x | x |  |  | x | x |
| **D20S106** | **1** | x | x | x | x | x | x | x |  | x | x |  |  | x | x | x | x | x | x | x | x | x |  | x | x |  |  | x | x |
| **D20S107** | **6** | x | x | x | x | x | x | x |  |  | x |  |  | x | x | x | x | x | x | x | x | x |  |  | x |  |  | x | x |

| **Markers** | **Common haplotype in full** | **3** | | | | | | | **4** | | | | | **5** | | | | | | | **6** | | | | | |
| --- | --- | --- | --- | --- | --- | --- | --- | --- | --- | --- | --- | --- | --- | --- | --- | --- | --- | --- | --- | --- | --- | --- | --- | --- | --- | --- |
|  |  | **o** | **o** | **o** | **o** |  | **o** | **o** | **o** |  |  |  | **o** | **o** | **o** | **o** | **o** | **o** | **o** |  | **o** |  | **o** |  |  |  |
| **D20S98** | **5** | x | x | x | x | x | x | x | x | x | x | x | x | x | x | x | x | x | x | x | x |  | x |  |  |  |
| **D20S118** | **3** | x | x | x | x | x | x | x | x | x | x | x | x | x | x | x | x | x | x | x | x |  | x |  |  |  |
| **D20S114** | **1** | x | x | x | x | x | x | x | x | x | x | x | x | x | x | x | x | x | x | x | x |  | x |  |  |  |
| **D20S48** | **3** | x | x | x | x | x | x | x | x | x | x | x | x | x | x | x | x | x | x | x | x | x | x | x | x | x |
| **D20S605** | **2** | **x** | **x** | **x** | **x** | **x** | **x** | **x** | **x** | **x** | **x** | **x** | **x** | **x** | **x** | **x** | **x** | **x** | **x** | **x** | **x** | **x** | **x** | **x** | **x** | **x** |
| **D20S182** | **3** | **x** | **x** | **x** | **x** | **x** | **x** | **x** | **x** | **x** | **x** | **x** | **x** | **x** | **x** | **x** | **x** | **x** | **x** | **x** | **x** | **x** | **x** | **x** | **x** | **x** |
| **M189K21** | **2** | **x** | **x** | **x** | **x** | **x** | **x** | **x** | **x** | **x** | **x** | **x** | **x** | **x** | **x** | **x** | **x** | **x** | **x** | **x** | **x** | **x** | **x** | **x** | **x** | **x** |
| **D20S139** | **5** | x | x | x | x | x | x | x | x | x |  |  | x | x | x | x | x | x | x | x | x | x | x | x | x | x |
| **D20S190** | **5** | x | x | x | x | x | x | x | x | x |  |  | x | x | x | x | x | x | x | x | x | x | x | x | x | x |
| **D20S106** | **1** | x | x | x | x | x | x | x | x | x |  |  | x | x | x | x | x | x | x | x | x | x | x | x | x |  |
| **D20S107** | **6** | x | x | x | x |  | x | x | x |  |  |  | x | x | x | x | x | x | x |  | x | x | x | x | x |  |

| **Markers** | **Common haplotype in full** | **7** | | | | **8** | | | | **9** | | **10** | | **11** | **12** |
| --- | --- | --- | --- | --- | --- | --- | --- | --- | --- | --- | --- | --- | --- | --- | --- |
|  |  |  | **o** | **o** | **o** |  |  | **o** | **o** |  |  |  |  |  | **o** |
| **D20S98** | **5** | x | x | x | x |  |  | x | x | x | x |  |  |  | x |
| **D20S118** | **3** | x | x | x | x | x | x | x | x | x | x |  |  |  | x |
| **D20S114** | **1** | x | x | x | x | x | x | x | x | x | x | x |  |  | x |
| **D20S48** | **3** | x | x | x | x | x | x | x | x |  |  | x |  |  | x |
| **D20S605** | **2** | **x** | **x** | **x** | **x** | **x** | **x** | **x** | **x** | **x** | **x** | **x** | **x** | **x** | **x** |
| **D20S182** | **3** | **x** | **x** | **x** | **x** | **x** | **x** | **x** | **x** | **x** | **x** | **x** | **x** | **x** | **x** |
| **M189K21** | **2** | **x** | **x** | **x** | **x** | **x** | **x** | **x** | **x** | **x** | **x** | **x** | **x** | **x** | **x** |
| **D20S139** | **5** | x | x | x | x | x | x | x | x | x | x | x | x | x | x |
| **D20S190** | **5** | x | x | x | x |  |  | x | x |  |  |  |  |  | x |
| **D20S106** | **1** | x | x | x | x |  |  | x | x |  |  |  |  |  | x |
| **D20S107** | **6** |  | x | x | x |  |  | x | x |  |  |  |  |  | x |

| **Markers** | **Common haplotype in full** | **15** | | | | | | | **16** | | **17** |
| --- | --- | --- | --- | --- | --- | --- | --- | --- | --- | --- | --- |
|  |  |  |  |  |  |  |  |  |  |
| **D20S98** | **5** |  |  |  |  | x | x | x | x | x |  |
| **D20S118** | **3** |  |  |  |  |  | x | x |  | x | x |
| **D20S114** | **1** |  | x |  |  | x |  |  | x |  |  |
| **D20S48** | **3** |  | x |  |  | x | x |  | x | x |  |
| **D20S605** | **2** |  |  |  | x | x |  |  | x | x | x |
| **D20S182** | **3** | x | x |  |  | x |  |  | x | x | x |
| **M189K21** | **2** | x | x | x | x | x | x | x |  |  |  |
| **D20S139** | **5** | x | x | x | x | x |  |  | x | x | x |
| **D20S190** | **5** |  |  |  |  | x |  |  | x | x |  |
| **D20S106** | **1** |  |  |  |  |  |  | x |  |  |  |
| **D20S107** | **6** |  |  | x |  |  |  |  |  |  | x |
